# Is blood-brain barrier a probable mediator of non-invasive brain stimulation effects on Alzheimer’s disease?

**DOI:** 10.1038/s42003-023-04717-1

**Published:** 2023-04-14

**Authors:** Aleksandra Petrovskaya, Artem Tverskoi, Angela Medvedeva, Maria Nazarova

**Affiliations:** 1 Engelhardt Institute of Molecular Biology, Russian Academy of Sciences, Moscow, 119991 Russia; 2 Department of Chemistry, Rice University, Houston, TX 77005 US; 3 Athinoula A. Martinos Center for Biomedical Imaging, Department of Radiology, Massachusetts General Hospital, Harvard Medical School, Charlestown, MA 02129 USA; 4 Center for Cognition and Decision Making, Institute for Cognitive Neuroscience, National Research University Higher School of Economics, Moscow, 101000 Russian Federation

**Keywords:** Blood-brain barrier, Alzheimer's disease

## Abstract

Alzheimer’s disease (AD) is a complex neurodegenerative disease with no existing treatment leading to full recovery. The blood-brain barrier (BBB) breakdown usually precedes the advent of first symptoms in AD and accompanies the progression of the disease. At the same time deliberate BBB opening may be beneficial for drug delivery in AD. Non-invasive brain stimulation (NIBS) techniques, primarily transcranial magnetic stimulation (TMS) and transcranial direct current stimulation (tDCS), have shown multiple evidence of being able to alleviate symptoms of AD. Currently, TMS/tDCS mechanisms are mostly investigated in terms of their neuronal effects, while their possible non-neuronal effects, including mitigation of the BBB disruption, are less studied. We argue that studies of TMS/tDCS effects on the BBB in AD are necessary to boost the effectiveness of neuromodulation in AD. Moreover, such studies are important considering the safety issues of TMS/tDCS use in the advanced AD stages when the BBB is usually dramatically deteriorated. Here, we elucidate the evidence of NIBS-induced BBB opening and closing in various models from in vitro to humans, and highlight its importance in AD.

## Abstract (web summary)

Evidence from in vitro experiments, animal models and humans is revisited and highlights the potential role of the blood-brain barrier in mediating the effects of non-invasive brain stimulation in AD.

## Introduction

Alzheimer’s disease (AD) debilitates a large number of older individuals worldwide, and the affected population is increasing due to greater human longevity, incurring enormous costs on treatment and palliative care^1^. Usually initial symptoms of AD, such as memory loss and slower response times are recognized only when tremendous changes in the brain have already occurred, that is why the therapeutic strategies usually target highly affected brain^2,3^. Several pharmacological approaches are currently applied as prospective tools for AD treatment, however, the drug therapy is still far from being highly effective in AD^4–7^. Non-Invasive Brain Stimulation (NIBS) techniques have been tested both as an alternative and an addition to pharmacological approaches in AD^8,9^. In recent years, the number of efforts to shed light on NIBS mechanisms in AD increased substantially (see for the review^10^). In the majority of these studies, primarily neural mechanisms were targeted. However, AD is characterized not only by the neural mechanisms^2,3^ but also by non-neuronal changes including the substantial blood-brain barrier (BBB) breakdown^11,12^. At the same time, there are ongoing clinical trials (https:// clinicaltrials.gov/ [12/21/2022]) aiming at the BBB opening by focused ultrasound stimulation (FUS) for better drug delivery in AD. In AD, the most used NIBS approaches are transcranial magnetic stimulation (TMS) and direct current stimulation (tDCS)^10,13^, and their effects on the BBB have not been studied extensively yet. We argue that studies of TMS/tDCS effects on the BBB in AD are necessary to better understand their mechanisms and to boost their effectiveness in AD. Moreover, such studies are important considering the safety issues of NIBS use in AD stages when the BBB is damaged. Here, we elucidate the evidence of NIBS-induced BBB changes in humans, animals, and cellular models and highlight its importance in AD.

## Blood–brain barrier role in Alzheimer’s disease: etiology, pathophysiology, and modeling

**Fig. 1:**
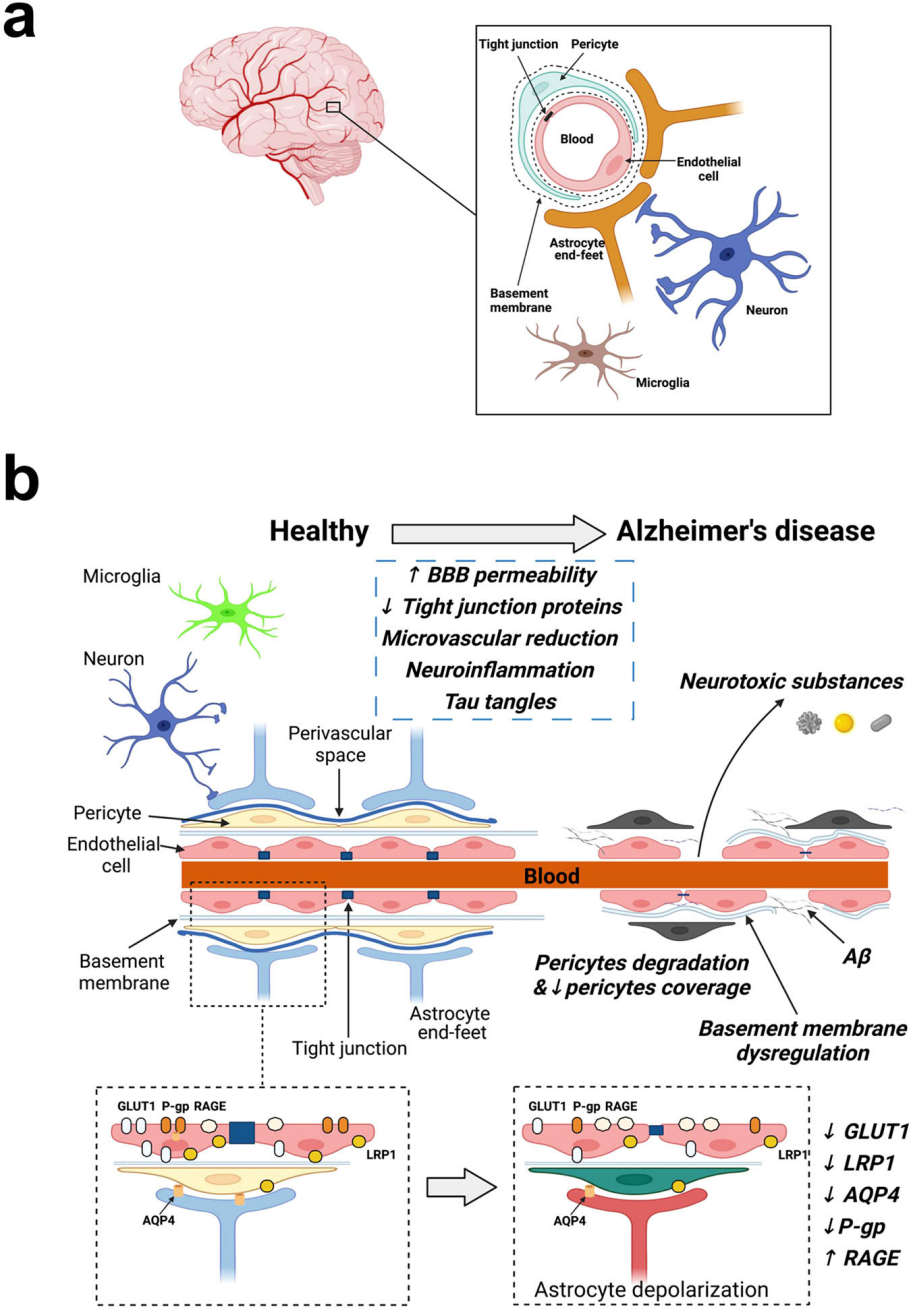
Blood-brain barrier in health and in Alzheimer’s disease. **a** The blood-brain barrier is composed of endothelial cells, forming tight junctions, pericytes, astrocytes, and neurons. **b** The blood-brain barrier (BBB) state in a healthy person (left) and in Alzheimer’s disease (AD) patient (right) is shown. At the top of the image, AD-associated BBB changes are shown, such as microvascular reduction, degradation of endothelial cells, loss of pericyte number, capillary coverage and capillary basement membrane rearrangement, etc. BBB damage activates the processes of neuroinflammation reflected by the peripheral macrophage and neutrophil infiltration. At molecular level (in the figure, below), protein homeostasis in health (left) and in AD (right) is given: decrease in aquaporin 4 (AQP4), low-density lipoprotein receptor-related protein 1 (LRP1), glucose transporter 1 (GLUT1), P-glycoprotein (P-gp) and increase in receptor for advanced glycation end products (RAGE) levels. For details, see refs. ^38,80,84^.

The BBB is a semi-permeable membrane within mature brain microvessels, protecting neurons from factors presenting in the systemic circulation. The BBB is formed by the components of the neurovascular unit—vascular cells (endothelial cells, pericytes, smooth muscle cells), glia (astrocytes, oligodendroglia, microglia), neurons and extracellular matrix^11,14,15^. Endothelial cells form tight junctions—a physical barrier, whereas other cell types provide signaling among the cells. The BBB breakdown in AD is an increase in vascular permeability associated with a decrease in the expression of tight junction proteins (Fig. 1; ref. ^16^). The BBB breakdown accompanies even healthy aging, while in patients with mild cognitive impairment and AD dementia, the BBB breakdown is accelerated^17^. The BBB-associated changes may be an early AD biomarker^18,19^. Notably, brain endothelial cells, among all vascular cells, demonstrate their higher vulnerability in AD, based on gene expression data^20^. Although there is no etiotropic treatment, the search for AD biomarkers at early stages is important^21^ because the early diagnosis may serve as a motivation to correct a lifestyle and decrease possibly modifiable risks. For example, positron emission tomography (PET) studies have already shown that patients with mild cognitive impairment have reduced glucose uptake across the BBB and, therefore, disintegrated BBB in young subjects may be considered an early marker of AD^22,23^. The BBB leakage is associated with changes in tight junctions, the overall BBB permeability index, transendothelial resistance, etc^24,25^., and all such alterations were reported in AD pathology (Fig. 1)^26,27^.

Considering the key role of the BBB in pharmacological brain therapy in general, there are already plenty of approaches allowing to assess the BBB permeability, such as (1) in vitro transwell models, allowing BBB permeability assessment to fluorescent tracers and transendothelial electrical resistance (TEER)^28–31^; (2) in vivo techniques like microdialysis, used for the analysis of the BBB permeability changes during or after an intervention (e.g., NIBS, drug, injection of a pathogenic AD-associated protein)^32^; (3) specific type of positron emission tomography: F-2-fluoro-2-deoxy-d-glucose-PET in humans^33,34^.

The BBB disruption and Aβ propagation seem to be intertwined, but they are mostly parallel processes. It is known that in AD, matrix metalloproteinases may digest tight junction and adherent proteins of the BBB^27^, decreasing the levels of tight junction proteins in the brain, and resulting in the physical breakdown of the BBB^26^. Aβ in AD also alters the tight junction proteins content, destroying the BBB^29,35^. Recent preliminary data have demonstrated that repetitive TMS (rTMS) simultaneously diminishes the matrix metalloproteases level and improves cognitive functioning in MCI patients^36,37^. AD-induced BBB abnormalities associated with changes in matrix metalloproteinase (MMP) and tight junction protein content are tightly connected with amyloid, and it makes these parameters sensitive to strong therapeutic stimuli. Brain stimulation is supposed to belong to a number of stimuli that may affect AD progression via the BBB disruption. Better understanding of the pathogenic proteins associated with AD—either peripheral or central—is needed for better understanding of the BBB role in AD. A collaborative work between clinicians and scientists is required to piece together knowledge on AD progression and the BBB breakdown.

## Non-invasive brain stimulation as a possible treatment in Alzheimer’s disease

Among techniques most widely used to target neuronal dysfunction in neurodegeneration, TMS and tDCS are in precise focus of the perspective. NIBS-mediated modulation of cognitive functions in AD is commonly considered to be connected with the changes in neural activity^13,38^, while vascular aspects are mostly overlooked. At the same time, vascular pathology in AD is highly amyloid-dependent considering (1) probable amyloid peripheral origin, (2) its high concentration in the brain blood flow, (3) co-localization of classic and diffuse amyloid plaques with the brain vessels, and (4) known biochemical and functional amyloid effects on the BBB cells^29,39–41^. Thus, NIBS amyloid targeting may be considered in a tight association with probable NIBS effects on the brain vascular system. Among attractive targets for TMS/tDCS application in AD are the frontal lobe, specifically, Broca’s area, dorsolateral prefrontal cortex, parieto-temporal lobe (Wernicke’s area), bilateral parietal somatosensory association cortices, temporal lobe^42,43,44,45^.

tDCS is a safe and easy-applicable method, which is widely studied in AD. Different tDCS protocols applied in AD patient cohorts improved recognition memory and general cognitive abilities, but the results were variable among studies^45–47^. A randomized, placebo-controlled study in AD patients revealed no effect of temporal cortex tDCS on cognition^48^, while the studies confirming temporal lobe tDCS effects in AD differed in study design, protocols and cognitive tasks^45,49^.

TMS is another widely used NIBS approach with better spatial accuracy compared to tDCS. The use of TMS at the earlier stages of AD was demonstrated as a prospective tool for treatment^38,50^. According to Dong and co-authors’ review^42^, TMS modulates cognitive functions based on the Alzheimer’s Disease Assessment Scale-Cognitive Subscale (ADAS-cog), but does not affect Mini-Mental State Examination (MMSE) score. The review articulates the importance of TMS frequency in AD treatment: high-frequency rTMS shows big effect, at least based on ADAS-Cog scale. Multiple TMS/tDCS studies both in patients and in healthy participants were followed by their unsuccessful replications^51–54^. We hypothesize that replication failures related to TMS/tDCS application in AD may be in part connected with our poor understanding of their non-neuronal effects, including effects on the BBB.

## Possible influence of transcranial magnetic and electric stimulation on blood-brain barrier in Alzheimer’s **disease**

Here, we focus on the BBB as a probable mediator of NIBS effects in AD. The BBB-mediated random NIBS effects may be connected with changes in tight junction protein expression, transendothelial resistance, permeability to molecules, etc. We suggest that in AD, TMS/TES-induced non-neuronal changes may be an important mediator of TMS/TES effects. Apart from the immediate changes of the BBB permeability, there is evidence of the endothelial intracellular parameters’ change, including mitochondria abnormalities and FGF-2-related changes^55^. Interestingly, magnetic field effects on endothelial cells may be associated with the same factors^56–58^. Electric field effects on endothelial cells are mediated by vascular endothelial growth factor (VEGF) receptor signaling, activation of ATP-receptor P2Y, Ca^2+^and NO concentration transients^59–61^. These factors are highly involved in AD pathology^62–64^. Importantly, in AD mouse model, BBB endothelial cells produce a variety of factors, such as thrombin, vascular endothelial growth factor (VEGF), angiopoietin-2, tumor necrosis factor (TNF), transforming growth factor, interleukin (IL) IL-1, IL-6, IL-8, monocyte chemoattractant protein-1, hypoxia-inducible factor-1, MMPs, and integrins that may promote AD pathogenesis^65^. A systematic analysis of NIBS effects on AD-associated factors in endothelial cells has not yet been conducted, but some data regarding neurons and glial cells are already available. It was reported, for example, that 10 Hz rTMS may significantly modulate anti- and proinflammatory marker expression either in primary astrocytes exposed to oxygen-glucose deprivation/ reoxygenation and make a neuroprotective effect^66^. For instance, the expression of IL-10 gene, associated with anti-inflammation, significantly increased in the astrocyte culture after 10 Hz rTMS^66^. Thus, as interleukin-10 is a supporting factor for the endothelium^67^, we hypothesize that NIBS might induce both direct and indirect effects on the endothelial cells, at least within a neurovascular unit. All this legitimates the concerns about the necessity to test the BBB integrity before/after TMS/TES application in AD. As the BBB state and AD progression are connected^11,68^, we hypothesize that in the earlier stages of AD, TMS/TES is applied to a brain with a less damaged BBB. In contrast, at later stages, TMS/TES influences already a not-integrative BBB (Fig. 1). Thus, for the early AD stages, the possibility of short-term NIBS-induced BBB opening may be helpful for drug delivery, while for the later AD stages, BBB restoration can be considered a goal, and an excessive BBB opening is a safety issue. Here, we discuss the experimental works dedicated to the effects of magnetic (Table 1) and electric (Table 2) stimulation on endothelial cell cultures and the BBB cells, in vivo animal models, and in humans and argue for their relevance for TMS/TES neuromodulation in AD.

**Table 1:** Examples of the magnetic stimulation effects on the BBB in different models: cellular, animal and human studies.

Biological level/model	Relevant effects	Stimulation protocol				Possible influence on the BBB	Reference
		Method	Conditions				
			Key stimulation parameters	Area	Sample		
Human studies	Increase in barrier permeability to Gadolinium-based contrast agent after deep TMS; the effect is prevented when neuronal activity is blocked but not under glutamate application	deep TMS	1 Hz; pulse duration, 360 μs; train duration, 50 s; intertrain interval, 60 s; the number of trains, 5; the total number of pulses, 250; stimulation intensity, 130% of RMT.	The anterior periphery of the resected tumor bed	15 individuals with glioblastoma and 3 males as healthy control: a pilot study	Increase in the BBB permeability	^73^
Animal studies	No significant changes in permeability surface-area product (PS) for the tracers	pulsed TMS	1 magnetic stimulation every 10 s for 10 min	Ventrally and dorsally on the cranium	10 Wistar rats	No effect on the BBB integrity	^72^
			50 magnetic stimulations (each stim. -10 min) a day for one week		10 Wistar rats		
			Control, no stimulation		10 Wistar rats		
	Decrease in: leakage of FITC-Dextran, IgG intensity, relative leakage of Evans blue, cytokine response, apoptosis of endothelial cells; excessive astrocyte-vasculature interactions. Improvement and increase in ZO-1, occludin, claudin-5, and the endocytotic scaffolding; protein caveolin-1, collagen IV, and MMP9 at day 6 after stroke; tissue oxygenation, parameters of vascular structure and morphology, blood perfusion, MMP2 at day 22, VEGF expression; TGFβ expression; levels of PDGFRβ associated with A2 astrocytes and their adjacent vasculature; vessel-associated expression of HIF-1α. All the results reveal changes after exposure of photothrombotic stroke rats to rTMS compared to the photothrombotic stroke rat model.	continuous theta-burst rTMS	Five-min theta-burst rTMS treatment (3 pulses of 50 Hz, repeated every 200 ms); 3 h after PT stroke and administered daily from day 1 to day 6 after stroke; intensity 200 Gauss	Infarcted hemisphere	Photothrombotic (PT) rat model: a control group and a group with PT stroke and sham rTMS and a group with PT stroke and rTMS (+ in some cases, healthy control)	Increase in the BBB integrity	^80^
	Increase in vascular permeability: enhancement via 1 Hz TMS, but not significant with 10 Hz stimulation.	rTMS	low frequency: 1 Hz, 50 μs pulse duration; duration, 50 s train; intertrain interval, 60 s; number of trains, 5; total number of pulses, 250 and high frequency: 10 Hz, 50 μs pulse duration; train duration, 1 s; intertrain interval, 9 s; number of trains, 5; trains were repeated 5 times with 60 s intervals; total number of pulses, 250. Stimulation intensity was set to 130% of resting motor threshold.	No specificity for stimulation	Sprague Dawley rats, thrombotic stroke model	Increase in the BBB permeability	^73^
	Increase in permeability to fluorescein tracer, NaFluo, within min after 1 Hz rTMS onset, which gets normalized at 15-30 min from TMS; cortical vascular permeability to IGF-Trap after 1 Hz rTMS. Decrease in permeability to fluorescein tracer, NaFluo, within min at 120 min after rTMS onset.	rTMS	1 Hz, 130% of resting motor threshold, with 360 µs pulse duration, 50 s train duration, 60 s inter-train interval, 5 trains, and 250 pulses in total. Total session duration was ~8.2 min.	Top of the rat’s head. One side of the coil’s outer perimeter was placed on top of the right hemisphere, perpendicular to the posteroanterior midline of the cranium, with the contralateral side elevated from the head.	Sprague–Dawley rats	the BBB opening and no brain injury after 1 Hz rTMS	^74^
Cell culture studies	Increase of 40% in cell number after exposure to 120 μT static magnetic fields; increase in cell number after exposure to 60 μT magnetic field for 24 h per day but not 1 hour a day. Increase in eNOS positive cell number after 3 days of 120 μT magnetic treatment. No changes in VEGF expression ratio after 24 h and 48 h exposure to magnetic field. No changes in NO concentration after stimulation for 30 min, 2 h, 5 h, and 24 h.	static magnetic field	60 or 120 μT field intensity for 24 h per day; 60 μT for 1 h per day		HUVEC	Increase in proliferation of endothelial cells and eNOS expression. Limitation: HUVECs are not the BBB cells.	^82^

**Table 2:** Examples of the electric stimulation effects on the BBB in different models: cellular, animal and human studies.

Biological level / model	Relevant effects	Stimulation protocol				Possible influence on BBB	Reference
		Method	Conditions				
			Stimulation parameters	Area	Sample		
Human studies	No pathological alterations after tDCS treatment in 30 and 60 min after tDCS application based on MRI results.	tDCS	1 mA; 13 min for the anodal and 9 min for the cathodal motor cortex stimulation.	One electrode –above C3 (the left motor cortex), the other electrode – above the contralateral orbit (the right frontopolar cortex).	10 healthy individuals. The anode is over motor cortex (5 subjects); the anode – over contralateral orbit (5 subjects).	No BBB damage	^77^
Animal studies	Increase in permeability to sodium fluorescein and FITC-Dextran at 5,10 and 15 min after tDCS application. At 20 min permeability returns to the control. tDCS mechanism is NO dependent: NOS inhibition ameliorates tDCS-induced permeability to fluorescent tracers at 5,10,15 and 20 min post-tDCS treatment.	tDCS	3 different doses of weak direct current (0.1, 0.5, and 1 mA) for 20 min (including 30 s ramp up and 30 s ramp down)	Epicranial anode electrode – over the right or left frontal cortex of a rat head (~2 mm anterior to Bregma and 2 mm right to Sagittal suture); the returning electrode - onto the ventral thoracic region.	Sprague Dawley rats	Increase in BBB permeability	^93^
	Increase in BBB permeability and solute diffusion coefficient in rat brain tissue by transiently enhancing the width of the brain extracellular matrix and reducing its density.	tDCS	20 min 0.1–1 mA of anodal direct current to rat brain using epicranial electrodes	right frontal cortex of rat (~2 mm posterior to bregma and 2 mm right to sagittal suture)	Sprague Dawley rats	Increase in BBB permeability	^93^
Cell culture studies	1 mA/10 min DCS does not damage ZO-1. Increase in water flux, convective and diffusive permeability to 70 kDa tetramethylrhodamine isothiocyanate, but not to 5-Carboxytetramethylrhodamine-430Da after 1 mA/10 min DCS. Post-DCS effect is insignificant compared to the control.	Direct current stimulation	0.1–1.5 mA current across the monolayers for 10 min		bEnd.3	Possible BBB permeability increase for large but not small substance. No damage to tight junction protein ZO-1.	^84^
	Decrease in TEER after 0.5 and 1 mA/cm^2^ for 5 and 10 min in hCMEC; No TEER changes after 0,1 mA/cm^2^ for 5 or 10 min in hCMEC. Increase in permeability to FITC-Dextran after 0,5 mA/cm^2^ (10 min) and 1 mA/cm^2^ for 5 and 10 min; no changes after 0,5 mA/cm^2^ for 5 min in hCMEC. Decrease in heparan sulfate intensity after 0,5 mA/cm^2^ (10 min) and 1 mA/cm^2^ for 5 and 10 min; no changes after 0,5 mA/cm^2^ for 5 min and 0.1 mA/cm^2^ for 5 and 10 min in hCMEC. Decrease in intensity of hyaluronic acid after 1 mA/cm^2^ for 10 min in hCMEC. The effects on heparan sulfate and TEER in bEnd.3 were similar to the ones in hCMEC. Disruption of ZO-1, endothelial glycocalyx by DCS is NO dependent. Decrease in TEER after 1 mA/cm^2^ for 10 min in bEnd.3. Decrease in ZO-1 concentration distribution after 1 mA/cm^2^ for 10 min. Disruption of Tight Junctions by DCS is NO dependent. Increase in TEER after NOS inhibition for 60 min in hCMEC. Decrease in permeability to Dextran-70 kDa after NOS inhibition for 60 min in hCMEC. Neutralization of DCS effects on TEER and permeability to Dextran-70 kDa after 60 min – pretreatment of 1 mM L-NMMA.	Direct current stimulation	0.1–1 mA/cm^2^ for 5 or 10 min	Anode – on the upper chamber 7 mm above the in vitro BBB, cathode – in the bottom chamber 7 mm below the in vitro model.	bEnd.3 & hCMEC/D3 or hCMEC	Increase in BBB permeability	^94^

### NIBS effects on blood-brain barrier opening

NIBS-induced BBB opening may be promising for drug delivery in AD, and it has already been tested for the hippocampus^69,70^ and prefrontal cortex^71^ using FUS. At the same time, BBB opening may be a safety concern in AD. Thus, one should test whether TMS/tDCS-induced BBB opening is not associated with pathological features of the BBB deterioration characteristic for AD (e.g. pericyte, endothelial, and neuronal degeneration; the BBB transporter function abnormalities; inflammation; accumulation of toxic agents)^11^. The TMS/tDCS effects on BBB opening are summarized below. The effect of magnetic stimulation on the BBB-associated mechanisms was investigated from the very beginning of TMS use. In the early 1990 study, Ravnborg and co-authors showed that single-pulse TMS in a rat did not change the BBB permeability^72^. However, since that time, there were several successful efforts to open the BBB using TMS^73,74^. TMS increased BBB permeability both in animals (in rats, at a low frequency but not at a high frequency) and in patients with malignant brain tumors^73^. Recently, in an attempt to trigger the BBB opening in a rat model, repetitive low-frequency TMS was applied to the rat’s right hemisphere, and fluorescent angiography revealed ~ 18% increased permeability 15 min after the stimulation onset. After the other 15 min, vascularization gets normalized^74^. It is worth noting that to be effective, NIBS-induced BBB opening interventions should be developed considering the pharmacokinetics, as the peak concentration of a drug in the brain may be achieved only in 2–3 h after the drug administration^75^. Also, there is significant evidence to support that tDCS may also modulate BBB integrity (see the review^76^). It was reported that tDCS temporarily increased the BBB permeability in a rat brain, and this effect was mediated by NO^61^. Nitric oxide synthase (NOS) inhibitor ameliorated tDCS-induced BBB permeability^61^. To our knowledge, there is the only human study, investigating BBB changes after 1 milliampere (mA) tDCS in healthy participants, which did not reveal any BBB changes using diffusion-weighted MRI^77^.

### NIBS effects on blood–brain barrier closure

Interestingly, there are also data supporting the BBB closing effect of NIBS. For instance, in the recent rTMS work on a rabbit eye model, it was shown that the expression of a tight junction protein ZO-1 increased after repetitive magnetic stimulation^78^. The main limitation of this study is that stimulation was applied to the cornea of a rabbit keratopathy model and, thus, targeted corneal epithelial barrier functions, not the BBB. However, the corneal epithelial and endothelial tissues in the brain vessels are similar in terms of ZO-1 expression, which may indirectly indicate that TMS might also strengthen the BBB in certain circumstances. Another study using electroconvulsive therapy on the Gunn rat model showed an increase in tight junction protein claudin-5 expression and astrocytic coverage of the brain blood vessels after stimulation^79^. The limitation of this study is that one may not directly compare electrical, magnetic, and electroconvulsive therapy due to the different principles behind them. In the other recent work on the photothrombotic stroke rat model, the BBB damage was successfully mitigated by theta-burst TMS: BBB-associated tight junction protein expression, morphology, and perfusion of vessels preserved^80^. However, the direct comparison of the BBB modulation mechanisms in stroke and in AD is challenging, as the BBB disruption may be triggered by different factors^81^. To our knowledge, there are no studies directly reporting that NIBS in AD will lead to the BBB closure or may hamper the transport of pharmacotherapeutic agents to the brain.

There are also a few studies trying to mitigate the BBB disruption using TMS/TES where no clear effect on the BBB was shown. In vitro, endothelial cells HUVEC showed a change in their morphology and increased in number after exposure to static magnetic fields for 24 h a day; however, no effect was detected after shorter stimulation^82^. Although HUVEC cell line is not a BBB line, it is successfully used in BBB modeling^83^. An important marker of the BBB integrity—tight junction proteins expression - was not evaluated, while endothelial nitric oxide synthase (eNOS) expression and nitric oxide (NO) concentration—other sensitive markers in AD—were analyzed. eNOS expression increased after long-term exposure to the magnetic field, while NO level did not change^82^. In another study, to model the effect of tDCS on the BBB functions, a direct electrical current was applied to endothelial cells bEnd.3^84^. Anti-ZO-1 immunostaining of the cells demonstrated no damage to the cell monolayer integrity^84^. Probably, the intracellular parameters (e.g., NO, Ca^2+^) are more sensitive than physiological parameters (e.g., transendothelial electrical resistance and BBB permeability to fluorescent tracers), however, the last ones are more significant as markers for the assessment of the NIBS effect.

We can conclude that the majority of the NIBS effects on BBB in the non-pathological model were toward BBB opening. In those studies when the BBB integrity was increased, pathological models were used^85–87^.

## Discussion

There are many blind spots in AD pathology, which result in difficulties in the formulation of a desirable therapeutic effect of NIBS on the BBB. To answer this question, a translational approach is needed. However, translational studies of NIBS effects on the BBB in AD are challenging because, on the one hand, BBB studies are difficult in humans, and on the other hand, NIBS studies are difficult in in vitro and in vivo models. Lack of understanding of non-neuronal NIBS mechanisms in AD leaves multiple questions unanswered. Should one aim at BBB opening or closing in AD patients depending on the AD stage? Is the BBB state associated with the changes in cognitive performance? Is NIBS-induced BBB opening similar to the mechanism of the BBB disruption in neurodegeneration? Is NIBS effect in AD similar to NIBS effect in other conditions where BBB is compromised, such as multiple sclerosis^11,88,89^, Diabetes Mellitus^90^, etc. We believe that more studies should address the question of NIBS effects on the BBB as in AD, as well as in healthy aging. We believe that the parameters of a model should be chosen with an emphasis on its sensitivity to reveal possible fine NIBS effects on the BBB. We summarize candidate parameters for the future BBB investigation in cells, animals, and humans, in Table 3. We argue that in order to extrapolate the results of in vitro and in vivo animal works to human participants, one has to study NIBS effects on key components of AD pathology such as extra- and intracellular ion concentration changes, transendothelial resistance, and the proteins, primarily, Aβ and tau. At the same time, NIBS effects on transendothelial resistance and other in vivo parameters should be probed not only in intact animals but also in AD animal models. Yet another point for the NIBS effects on the BBB in AD is that additional investigation may be needed to assess NIBS-induced BBB mitigation in advanced AD stages for safety reasons. Considering that BBB mitigation using transcranial ultrasound stimulation (TUS) is already performed in clinical studies^91,92^, we believe that the approaches used for the BBB assessment in such studies may be extrapolated to TMS/TES studies.

**Table 3:** «Q*uo vadis*»: possible future directions for the BBB investigation after NIBS treatment.

Cell culture studies			Animal studies		Human studies	
**Parameters to be analyzed after electrical, magnetic or ultrasonic stimulation**						
• Viability • Proliferation • Morphology • Intracellular redox-sensitive parameters • Intracellular ion concentrations • NO • Permeability to tracers • Transendothelial electrical resistance • Distribution and level of the tight junction proteins			• Distribution and level of the tight junction proteins • Morphology • Permeability to tracers • Tracer localization in different brain areas • Intracellular redox-sensitive parameters		• The BBB permeability to tracers • Tracer localization in different brain areas • Pathomorphology	
**Approach (model)**						
Immortalized human brain endothelial cells	The BBB cellular models of AD comprised of (1) primary endothelial cells (2) endothelial cells, astrocytes, neurons and pericytes	Induced pluripotent stem cells differentiated into brain cells	Behavior and cognitive performance in AD animal model	Molecular and cellular aspects in AD animal models	PET and MRI in AD clinical trials	Combination of NIBS and PET/MRI in AD clinical trials

## Conclusion

**Fig. 2:**
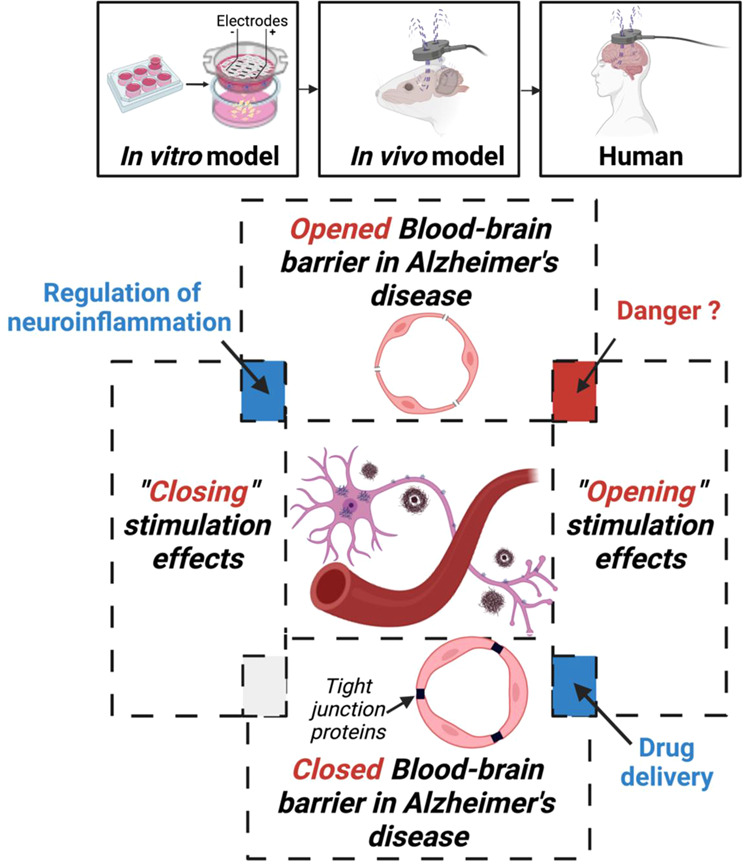
Scheme of the non-invasive brain stimulation (NIBS) action on blood-brain barrier (BBB) in Alzheimer’s disease (AD). To receive non-invasive brain stimulation representative data, translational approach can be used including human, animal, and cellular models.

NIBS techniques are widely tested as an alternative and an addition to pharmacological therapy in AD^8,9,69^. In this paper, we draw attention to the probable non-neuronal effects of NIBS such as BBB changes, both its opening and closing, and highlight its importance in AD. On the one hand, the BBB is vulnerable in AD, and its further deterioration might aggravate the pathology. On the other hand, BBB opening may be beneficial in AD in some cases for the purpose of drug delivery. We illustrate our point using evidence from human, animal, and cellular models (Fig. 2). The adaptation of NIBS protocols to *re-* and de novo investigating its effects on the BBB may be an important direction of the research in the field. We suggest that NIBS effects on the BBB should be investigated more considering that a large pool of cerebral blood vessels is located on the surface of the cortex, and is well accessible for NIBS.
